# Prediction of Potential Drug–Disease Associations through Deep Integration of Diversity and Projections of Various Drug Features

**DOI:** 10.3390/ijms20174102

**Published:** 2019-08-22

**Authors:** Ping Xuan, Yingying Song, Tiangang Zhang, Lan Jia

**Affiliations:** 1 School of Computer Science and Technology, Heilongjiang University, Harbin 150080, China; 2 School of Mathematical Science, Heilongjiang University, Harbin 150080, China

**Keywords:** drug–disease association, non-negative matrix factorization, projections of drug features, diversity representation, specific features of different drug views

## Abstract

Identifying new indications for existing drugs may reduce costs and expedites drug development. Drug-related disease predictions typically combined heterogeneous drug-related and disease-related data to derive the associations between drugs and diseases, while recently developed approaches integrate multiple kinds of drug features, but fail to take the diversity implied by these features into account. We developed a method based on non-negative matrix factorization, DivePred, for predicting potential drug–disease associations. DivePred integrated disease similarity, drug–disease associations, and various drug features derived from drug chemical substructures, drug target protein domains, drug target annotations, and drug-related diseases. Diverse drug features reflect the characteristics of drugs from different perspectives, and utilizing the diversity of multiple kinds of features is critical for association prediction. The various drug features had higher dimensions and sparse characteristics, whereas DivePred projected high-dimensional drug features into the low-dimensional feature space to generate dense feature representations of drugs. Furthermore, DivePred’s optimization term enhanced diversity and reduced redundancy of multiple kinds of drug features. The neighbor information was exploited to infer the likelihood of drug–disease associations. Experiments indicated that DivePred was superior to several state-of-the-art methods for prediction drug-disease association. During the validation process, DivePred identified more drug-disease associations in the top part of prediction result than other methods, benefitting further biological validation. Case studies of acetaminophen, ciprofloxacin, doxorubicin, hydrocortisone, and ampicillin demonstrated that DivePred has the ability to discover potential candidate disease indications for drugs.

## 1. Introduction

Developing a new drug is a complex, time-consuming, and expensive process [1,2], which typically proceeds through preliminary compound testing, pre-clinical and animal experiments, clinical research, and Food and Drug Administration (FDA) review, before it finally yields a new drug that reaches the market after 10–15 years, costing approximately 0.8–1.5 billion dollars [3,4,5,6]. Even with a substantial time commitment and capital investment, the successful development of a new drug is still associated with considerable risks [1,7,8]. Because the number of new drugs approved by the FDA has been declining since the 1990s [9,10], there is an urgent need to find alternative approaches that will reduce the development costs. Drug repositioning refers to the identification of new indications for drugs that have been approved by regulatory agencies. Compared to the development of a new drug for a certain indication, drug repositioning can shorten the drug development cycle to 6.5 years at the cost of approximately 0.3 billion dollars due to the known safety, tolerability, and efficacy profile of the drug candidate [11,12,13].

Computational prediction of new drug-related disease annotations can generate reliable drug–disease association candidates for further validation [14,15]. Previous prediction methods can be broadly divided into two categories. In first the category, the potential associations between drugs and diseases are usually related to shared target genes, and the more shared target genes there are, the higher the likelihood of a drug–disease association is. Therefore, several methods for predicting the association of drugs with diseases based on related target genes or gene expression profiles have been proposed [16,17]. Similarly, the possibility of a drug–disease association can be estimated based on the targeted protein complexes shared by the drugs and diseases [18] and the perturbed genes they have in common [19]. However, these methods are limited to drugs and diseases with shared genes or proteins.

The second category uses a variety of data types, including drug similarity, disease similarity, and target similarity, as well as interactions and association between drugs, targets, and diseases for drug repositioning. Wang et al. applied a kernel function to integrate similarity information drugs and diseases to predict potential drug–disease associations [20]. Several approaches integrate the information on drugs, targets, and diseases to create heterogeneous networks that infer drug candidates by information flow or random walks [21,22,23,24]. Some methods use the data of drugs and diseases to infer drug–disease association candidates using the logistic regression model [25], a statistical model [26], Laplacian regularized sparse subspace learning model [27], similar constraint matrix decomposition model [28] or non-negative matrix factorization model [29]. These methods include information from different sources and confirm that this information is important for predicting associations between drugs and diseases. However, multiple kinds feature of drugs, such as the chemical substructures and the target protein domains have diversity, and these methods did not take the diversity into account.

In this study, we present a new method, DivePred, for predicting potential drug–disease associations. DivePred deeply integrates not only the projection of multiple drug features in low-dimensional space but also the diversity of drug features. Projecting multiple high-dimensional drug features into the same dimension as the disease assists in measuring the distance between the drugs and the diseases, which is a critical parameter for the possibility of a drug–disease association. The chemical substructures of the drugs, the target protein domains, and the ontology annotation of the target gene, along with its associated disease annotations reflect the characteristics of the drugs from different perspectives. Therefore, retaining the diversity of multiple drug features can fully integrate information from different drug views. Thus, we created a unified model and developed an iterative optimization algorithm to derive drug–disease association scores. Experimental results based on cross-validation indicated that DivePred achieved better prediction performance than several state-of-the-art methods. Case studies of five drugs further demonstrated that DivePred could detect potential drug-related diseases.

## 2. Experimental Evaluation and Discussion

### 2.1. Evaluation Metrics

We used five-fold cross-validation to evaluate the performance of DivePred in predicting potential drug–disease associations. The known drug–disease associations were randomly divided into five equal subsets, four of which were used to train our model, while the remaining set was used to perform the test. In each cross-validation, $X^{\left(4\right)}$ contained only the drug–disease associations of the training set, and $R_{4}$ was calculated based on the known associations in matrix $X^{\left(4\right)}$. For a certain drug $r_{i}\left(1\leq r_{i}\leq N_{r}\right)$, its associated diseases in the test set was called the positive sample, and the other unmarked diseases were called negative samples. In the test results, a high positive sample rate of drug $r_{i}$ was correlated with an improved predictive performance for this drug.

A threshold θ was set, and when the score obtained by the sample estimate was higher than θ, it was identified as a positive example; otherwise, it was identified as a negative example. The TPRs (true-positive rates) and the FPRs (false-positive rates) under various θ can be calculated as follows,

(1) $$TPR=\frac{TP}{TP+FN},FPR=\frac{FP}{TN+FP}$$

where TP is the number of positive cases that were correctly identified, and TN indicates the number of negative examples that were correctly identified. FN and FP are the numbers of positive and negative examples that were misidentified, respectively. After calculating TPRs and FPRs for different θ values, the receiver operating characteristic curve (ROC) was be plotted. The area under the curve (AUC) was used as a measure to predict the performance of potentially associated disease with drug $r_{i}$. The overall performance of the prediction method was the average of the AUC values of all drugs.

Due to the imbalance of the number of positive and negative samples in the sample data, the precision–recovery rate (P–R curve) can provide additional information; precision and recall were defined as follows,

(2) $$precision=\frac{TP}{TP+FP},recall=\frac{TP}{TP+FN}$$

The precision ratio refers to the proportion of correctly identified positive samples in the search samples, and the recall rate is the same as the TPR. The area under the P–R curve (AUPR) was also used to measure the performance for predicting potential drug–disease associations.

Biologists typically choose the top-ranked candidates for further experimentation. It was our goal to increase the number of positive samples in the top-ranked section. To create another evaluation index, we calculated the recall rate of the top-ranked samples, which is the proportion of positive samples correctly identified in the top k of the list among the total of positive samples.

### 2.2. Comparison with Other Methods

To evaluate the performance of our prediction method, DivePred, we also compared it with several state-of-the-art methods for predicting potential drug–disease associations, including: TL_HGBI [21], MBiRW [22], LRSSL [27], and SCMFDD [28]. In our method of comparison, we need to fine-tune the hyperparameters. Based on five-fold cross-validation, we selected the hyperparameters values for $\alpha_{1},\;\alpha_{2},\;\alpha_{3},\;\alpha_{4}$ and $\alpha_{5}$ in DivePred from as $\left\{10^{-2},\;10^{-1},1,\;10,\;100\right\}$. DivePred achieved the best performance at $\alpha_{1}=1$, $\alpha_{2}=10$, $\alpha_{3}=\;$0.1, $\alpha_{4}=0.1,$ and $\alpha_{5}=0.1$. To perform a fair comparison with the four other methods, we used the best value provided by the authors to set the hyperparameters (i.e., α=0.4 and β=0.3 for TL_HGBI; α=0.3, l=2 and r=2 for MBiRW; μ=0.01, λ=0.01, γ=2, and k=10 for LRSSL; k=45%, μ=1 and λ=4 for SCMFDD).

**Figure 1:**
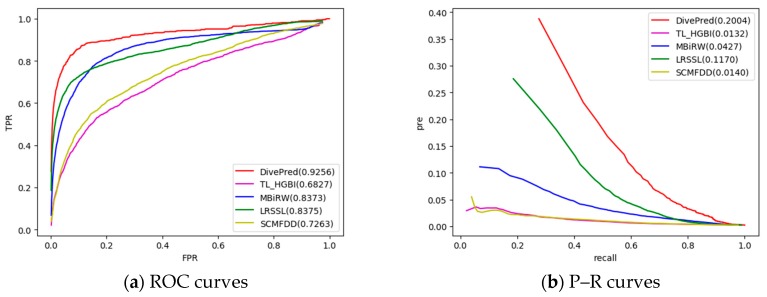
Two types of curves for evaluating the predicting performance of DivePred and other methods. (**a**) receiver operating characteristic (ROC) curves; (**b**) precision–recall (P–R) curves.

As shown in Figure 1a, DivePred achieved the best average performance, on a set of 763 drugs (AUC = 0.9256). Specifically, the performance score of DivePred was 24.29% better than that of the TL_HGBI algorithm, 8.83% better than the MBiRW algorithm, 8.81% better than the LRSSL algorithm, and 19.93% better than the SCMFDD algorithm. In addition, we tested 15 drugs using DivePred and the other four methods. The AUC values of the 15 drugs are shown in Table 1, DivePred preforms the best on 12 of these drugs. Among these comparison methods, LRSSL achieved a good performance because similar to DivePred, it considers the information on multiple drug features, although it does not consider the diversity of multiple feature information of the drugs. The MBiRW algorithm only considers a feature of the drugs, limiting its performance. The SCMFDD algorithm and TL_HGBI algorithm were relatively poor. The weak performance of the former might be due to the excessive dependence on the accuracy of similarity calculations; the latter may have problems due to the introduction of noise when calculating drug–drug similarity. Compared with those methods, DivePred was superior to those methods because it captures the specific features of each aspect of the drugs.

As shown in Figure 1b, the average PR curve of 763 drugs was higher for DivePred than those for the other methods, indicating that DivePred has the best performance for drug–disease association prediction (AUPR = 0.2004). Compared with the AUPR values of SCMFDD, TL_HGBI, MBiRW, and LRSSL, the DivePred values were 18.7%, 15.8%, 8.3%, and 18.6% higher, respectively. The AUPR values of the 15 drugs are shown in Table 2, and DivePred is the best performer on 10 of these drugs.

We evaluated the prediction results of 763 drugs by using a Wilcoxon test, and the results of the evaluation showed that DivePred was significantly better than other methods. These results were observed using a *p*-value threshold of 0.05, with DivePred showing better performance in terms of not only AUCs of ROC curves but AUCs of P–R curves as well (Table 3).

**Figure 2:**
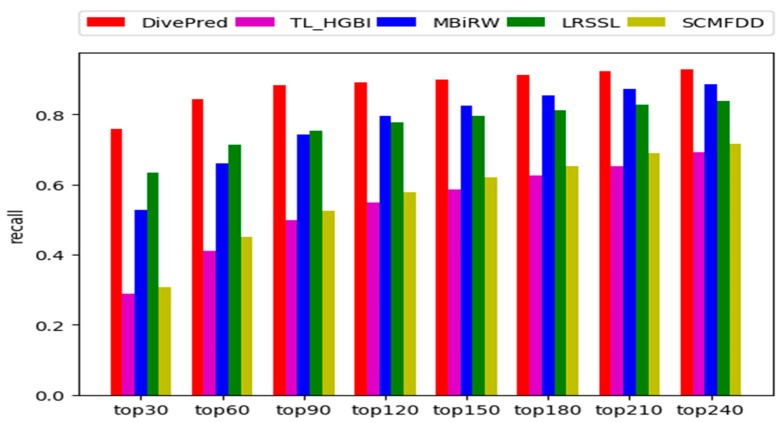
Average recall rates of all drugs at different top k.

In addition, the recall rates for the top k candidate diseases were assessed. A high recall rate for the top k candidate diseases indicated that the predictive method performed well in identifying diseases that are truly associated with a drug. The average recall rates of all 763 drugs at different top k values are shown in Figure 2. DivePred was always superior to the other methods in the range for of the top 30 to the top 240 candidates. Among the top 30, 90, and 150 candidate diseases, the recall rates for which were 74.6%, 87.4%, and 90.0%, respectively; the second-best method was LRSSL, where the recall rate was 63.4% in the top 30, 75.2% in the top 90, and 79.6% in the top 150; followed by MBiRW, for which the recall rates among the top 30, 90, and 150 candidates were 52.9%, 74.2%, and 82.6%, respectively; the worst performers were TL_HGBI and SCMFDD. Their recall rates were relatively close. For the former method, the recall rates were 28.8%, 49.6%, and 58.5% among the top 30, 90, and 150 candidate diseases, respectively. The recall rates for the latter method, SCMFDD, were 30.6%, 52.5%, 62.1% in the top 30, 90, and 150 respectively.

### 2.3. Case Studies on Five Drugs

To further demonstrate the ability of DivePred to discover candidate diseases for drugs, we conducted case studies on five drugs, including acetaminophen, ciprofloxacin, doxorubicin, hydrocortisone, and ampicillin. For each of the five drugs, we scored the drug–disease association predictions and ranked them accordingly. The top 15 diseases with the highest association scores were considered candidate diseases for the drug. A total of 75 candidate diseases were predicted, as shown in Table 4.

Comparative Toxicogenomics Database (CTD) is a powerful public database that provides relevant drugs information and the effects of drugs on diseases; this information is compiled from published literatures. DrugBank database is supported by the Canadian Institutes of Health Research, the Alberta Innovats-Health Solutions and the Metabolomics Innovation Centre. It provides clinical trial information on the drugs, including the drugs and the diseases being tested. PubChem is an open chemical database supported by the National Institutes of Health (NIH), which contains from various data sources with many informational entries on drugs and diseases. As shown in Table 4, 38 drug–disease association information were included in the CTD, 12 association information were contained in the DrugBank, and 10 association information were recorded by PubChem, indicating that these candidate diseases are indeed associated with the corresponding drugs.

Secondly, ClinicalTrials.gov (https://clinicaltrials.gov/) is an online clinical trial database managed by the National Library of Medicine (NLM) and the Food and Drug Administration (FDA), which contains a large amount of clinical research information on various drugs and diseases. Four drug–disease association predictions matched entries in the ClinicalTrials database. In addition, two candidates were labelled with “literature”, indicating that there is literature supporting that the candidate disease is being treated with the corresponding drug.

In addition, the CTD database also contains potential associations from literature data, which we included as “inferred candidate by k literatures”, where k represents the number of documents reporting that a drug that could be associated with a disease according to the CTD. A total of five candidates were tagged, indicating that this drug is more likely to be associated with the corresponding disease candidates. Of the 75 candidates, four could not be confirmed by observational evidence; they were labelled as “unconfirmed”.

### 2.4. Prediction of Novel Drug–Disease Associations

After evaluating its prediction performance by cross-validation, case studies, and the Wilcoxon test, we applied DivePred to predict novel drug–disease associations. All the known drug–disease associations were utilized to train DivePred’s prediction model. High-confidence candidate diseases of drugs were obtained using DivePred. Results are listed in supplementary Table ST1_candidates.

## 3. Materials and Methods

### 3.1. Datasets for Drug–Disease Association Prediction

We obtained drug feature data, disease similarity data, and drug–disease association data from previous studies by Wang et al., which included 763 drugs and 681 diseases, and 3051 drug–disease associations. The initial data were sourced from several databases: The chemical substructures of the drugs were represented by the chemical fingerprints defined in the PubChem database [32]; the domain composition of the proteins targeted by the drugs was obtained from the InterPro database; the protein ontology characteristics (molecular functions and biological processes) of the target proteins were extracted from the UniProt database.

### 3.2. Representation of Multi-Source Data

Our primary goal was to predict and rank diseases potentially associated with drugs that are of interest to us. A non-negative matrix factorization model was established by integrating multiple data about drug features, drug similarities, disease similarities, and drug–disease associations. Drug $r_{i\;}$ and disease $d_{j}$ association scores can be computed using our model. The higher the association score, the more likely is an association between $r_{i}$ and $d_{j}$. Three characteristic information representations of drugs including chemical drug features form an 881-dimensional binary chemical substructure vector, represented by the feature matrix $X^{\left(1\right)}\in R^{881\times N_{r}}$, where $N_{r}$ is the number of drugs, ${\left({\left(X^{\left(1\right)}\right)}^{T}\right)}_{j}$ is the jth row of the transposed of $X^{\left(1\right)}$ that indicates the case where the drug $r_{j}$ contains various chemical substructures. The term ${\left(X^{\left(1\right)}\right)}_{ij}$ is 1 if $r_{j}$ has a chemical substructure $c_{i}$, or it is 0 otherwise. The 1426-dimensional target protein domain features are represented by matrix; similarly, the jth column of $X^{\left(2\right)}$ indicates whether drug $r_{j}$ is associated with each protein domain. Using the matrix $X^{\left(3\right)}\in R^{4447\times N_{r}}$ to represent the 4447-dimensional target gene ontology feature ${\left(X^{\left(3\right)}\right)}_{ij}$ indicates whether the protein targeted by drug $r_{j}$ has the ith gene ontology; if so, the term ${\left(X^{\left(3\right)}\right)}_{ij}=1$ applies or it is 0 otherwise.

**Figure 3:**
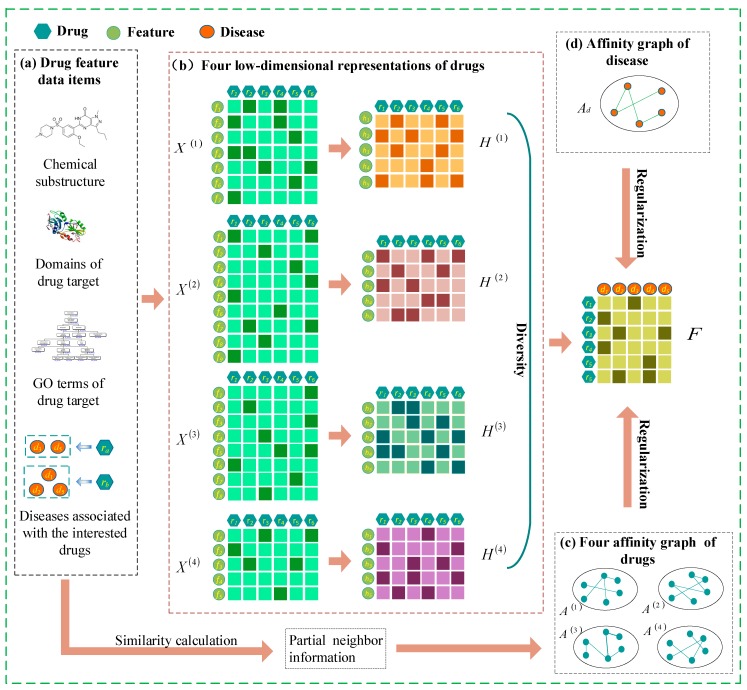
Representation of data from drugs and diseases from multiple sources and representation of drug–disease predictive association matrix F. (**a**) Drug feature data sets from multiple sources; (**b**) four low-dimensional representation of drugs; (**c**) four affinity maps of the drugs were obtained by similarity calculation; (**d**) extract the similarity of the diseases and obtain the affinity map of the disease.

**Calculation and representation of three types of drug similarities.** In this study, the similarity between drugs was assessed based on drug features and on the assumption that drug-related diseases are more likely to be similar when the drugs are more similar. For these three types of drug features, the more chemical substructures (or protein domains, or gene ontology attributes) are shared between two drugs, then the more similar they are (Figure 3a). Cosine similarity was computed to determine the similarity between drug $r_{i}$ and $r_{j}$ based on the three drug feature criteria, which are denoted as ${\left(R_{v}\right)}_{ij}$, where $R_{v}\in R^{N_{r}\times N_{r}}$ represents the similarity matrix of the vth feature data, v=[1, 2, 3]. Then, the cosine similarity was used to construct the similarity matrix of the vth drug feature,

(3) $${\left(R_{v}\right)}_{ij}=\frac{{\left(X_{v}\right)}_{i}\cdot {\left(X_{v}\right)}_{j}}{{∥(X_{v})∥}_{i}*∥{\left(X_{v}\right)}_{j}∥}$$

where ∥⋅∥ is the modulus of a vector.

**Calculation and representation of the fourth drug similarity.** From a previous publication, we used the drug–disease association data [17], and if two drugs are associated with more similar diseases, the more similar they are. We constructed the fourth drug feature matrix ${\left(X^{\left(4\right)}\right)}^{N_{d}\times N_{r}}$, where $N_{d}$ represents the number of diseases, and ${\left(X^{\left(4\right)}\right)}_{ij}$ is 1 if drug $r_{j}$ and disease $d_{i}$ are related or it is 0 otherwise. To compute the similarity feature matrix of the fourth criterion, $R_{4}\in R^{N_{r}\times N_{r}}$, we obtained the disease sets associated with drug $r_{i}$ and drug $r_{j}$ [33] and recorded them as $D_{i}=\left\{d_{1},d_{3}\right\}$ and $D_{j}=\left\{d_{2},d_{3},d_{5}\right\}$. The fourth similarity of $r_{i}$ and $r_{j}$ was calculated as follows,

(4) $${\left(R_{4}\right)}_{ij}=\frac{\sum_{a=1}^{m}\underset{1\leq b\leq n}{max}\left(D\left(d_{1a},d_{2b}\right)\right)+\sum_{b=1}^{n}\underset{1\leq a\leq m}{max}\left(D\left(d_{2b},d_{1a}\right)\right)}{m+n}$$

where $D\left(d_{1a},d_{2b}\right)$ is the semantic similarity between $d_{1a}$ belonging to $D_{i}$ and disease $d_{2b}$ belonging to $D_{j}$; m and n represent the number of diseases in $D_{i}$ and $D_{j}$, respectively. According to a previous study, Equation (4) calculates the semantic similarity between two diseases [33].

**Representation of the drug–disease association.** An association matrix $Y\in R^{N_{r}\times N_{d}}$ was established based on known drug–disease associations. Each row of Y corresponds to a drug, and each column corresponds to a disease. $Y_{ij}$ is 1 if there is a known association between drug $r_{i}$ and disease $d_{j}$ or it is 0 otherwise.

### 3.3. Drug–Disease Association Prediction Model

Our new predictive model, DivePred, merges various drug features and can be used to predict new indications for drugs. We know that if two drugs share more of the same features, they are more likely to have a high similarity, indicating a potential association with similar diseases, which is at the core of our new model.

**Modelling drug–disease association relationships.** We introduced the matrix $F=\left(F_{ij}\right)\in R^{N_{r}\times N_{d}}$ to represent the association score matrix of $N_{r}$ drugs and $N_{d}$ diseases to better describe the model. In the model, $F_{i}$ is the ith row of the association score matrix that represents the possibility of an association of drug $r_{i}$ with all diseases. $F_{ij}$ was the predicted association score between drug $r_{i}$ and disease $d_{j}$, and a high $F_{ij}$ indicates a stronger possibility of an association between $r_{i}$ and $d_{j}$. Since the non-zero elements in Y are very sparse, previous studies using sparse cases usually built optimizations based on observed relationships only [34,35,36]. Here, we assume that the known set of observed drug–disease association information is Ω, and the construct matrix is $M=\left(M_{ij}\right)\in R^{N_{r}\times N_{d}}$, where $M_{ij}$ was 1 if $\left(r_{i},d_{j}\right)\in \Omega$, or it is 0 otherwise (in fact, M=Y). All known related drug–disease pairs should also be included in the predictions, i.e., there are known associations drug–disease should have a higher score in the prediction results. Therefore, the squared loss function was defined as,

(5) $$min∥M⊙{\left(F-Y\right)∥}_{F}^{2}$$

where ${∥\cdot ∥}_{F}^{2}$ represents the Frobenius norm of a matrix, and ⊙ is the Hadamard product.

**Integrating multiple drug features into the model.** We replaced the original feature matrix with a new matrix obtained by non-negative matrix factorization to fuse different types of drug features. $X^{\left(v\right)}$ indicates the vth feature matrix of drugs, and a new drug feature matrix $H^{\left(v\right)}\in R^{N_{d}\times N_{r}}\left(1\leq v\leq 4\right)$ is obtained by matrix factorization of $X^{\left(v\right)}$ (Figure 3b); ${\left({\left(H^{\left(v\right)}\right)}^{T}\right)}_{i}$ is the ith row of the transposed of $H^{\left(v\right)}$, representing the new feature vector of drug $r_{i}$ in the vth view. While $W^{\left(v\right)}\in R^{d_{v}\times N_{d}}\left(1\leq v\leq 4\right)$ denotes the basic matrix of the vth drug feature, the jth row of $W^{\left(v\right)}$, ${\left(W^{\left(v\right)}\right)}_{j}$, indicates the weight of each new feature to the original jth feature. ${\left(W^{\left(v\right)}\right)}_{j}{\left({\left(H^{\left(v\right)}\right)}^{T}\right)}_{i}$ indicates the condition in which the drug $r_{i}$ has the original features $f_{j}$. To ensure that the new drug feature matrix represents the original feature matrix as much as possible, ${\left(W^{\left(v\right)}\right)}_{j}{\left({\left(H^{\left(v\right)}\right)}^{T}\right)}_{i}$ should match ${\left(X^{\left(v\right)}\right)}_{ji}$ as much as possible,

(6) $$\underset{H^{\left(v\right)},W^{\left(v\right)}\geq 0}{\min}∥M⊙{\left(F-Y\right)∥}_{F}^{2}+\alpha_{1}\overset{4}{\underset{v=1}{\sum }}∥X^{\left(v\right)}-W^{\left(v\right)}H^{\left(v\right)}{∥}_{F}^{2}\;$$

where $\alpha_{1}$ is a trade-off parameter that controls the weight of all drug feature information.

The multitude of drug similarities reflects the degree of similarity among the drugs from different aspects. There is consistency between the information from multiple aspects, but each view also has its own specific information. To ensure the diversity of each drug feature vector among the different views, we also require that each drug feature vector is as orthogonal as possible between the various views [37]. For example, $h_{i}^{\left(v\right)}$and $h_{i}^{\left(w\right)}$ are the representation vectors of the drug $r_{i}$ in the two drug feature views. To ensure that $h_{i}^{\left(v\right)}$ and $h_{i}^{\left(w\right)}$ are as different as possible, their dot product should approach zero.

(7) $$∥h_{i}^{\left(v\right)}\circ h_{i}^{\left(w\right)}∥{}_{1}=\overset{K}{\underset{j=1}{\sum }}h_{ji}^{\left(v\right)}\cdot h_{ji}^{\left(w\right)}$$

To derive a feature profile unique to every drug in each view, Formula (7) was introduced into the objective function.

(8) $$\underset{H^{\left(v\right)},W^{\left(v\right)}\geq 0}{min}∥M⊙{\left(F-Y\right)∥}_{F}^{2}+a_{1}\overset{4}{\underset{v=1}{\sum }}∥X^{\left(v\right)}-W^{\left(v\right)}H^{\left(v\right)}{∥}_{F}^{2}+a_{2}\overset{4}{\underset{w\neq v}{\sum }}tr\left(H^{{\left(v\right)}^{T}}H^{\left(w\right)}\right)$$

where $tr\left(H^{{\left(v\right)}^{T}}H^{\left(w\right)}\right)=\overset{N_{r}}{\underset{i=1}{\sum }}\overset{N_{d}}{\underset{j=1}{\sum }}h_{ji}^{\left(v\right)}\cdot h_{ji}^{\left(w\right)}$, and $a_{2}$ is used to control the contribution of the third term.

**Modelling the drug–disease association score.** In the drug–disease association score matrix *F*, the ith row of F, $F_{i}$, records the potential association score between drug $r_{i}$ and various diseases. Furthermore, $F_{i}$ is also the characteristic vector of $r_{i}$ at the disease level. The ith column of $H^{\left(v\right)}$, ${\left({\left(H^{\left(v\right)}\right)}^{T}\right)}_{i}$, is a new feature vector obtained after the original feature vector of the drug $r_{i}$ is projected onto the disease dimension. $H^{\left(v\right)}$ plays a guiding role in the assessment of drug–disease association scores, ${\left({\left(H^{\left(v\right)}\right)}^{T}\right)}_{i}$, and $F_{i}$ should be as consistent as possible. The extended objective function was defined as:

(9) $$\underset{H^{\left(v\right)},W^{\left(v\right)}\geq 0}{\min}∥M⊙{\left(F-Y\right)∥}_{F}^{2}+a_{1}\overset{4}{\underset{v=1}{\sum }}∥X^{\left(v\right)}-W^{\left(v\right)}H^{\left(v\right)}{∥}_{F}^{2}+a_{2}\overset{4}{\underset{w\neq v}{\sum }}DIVE\left(H^{\left(v\right)},H^{\left(w\right)}\right)\;+a_{3}\overset{4}{\underset{v=1}{\sum }}∥F-H^{{\left(v\right)}^{T}}{∥}_{F}^{2}\;$$

where $\alpha_{3}$ is the super-parameter that regulates the contribution of drug characteristic information throughout the model.

**Modelling the smoothness term.** Drug $r_{i}$ and its k neighbours are more likely to be associated with similar diseases. Hence, we established corresponding maps based on the drug neighbour information derived from the similarity of the four drugs. The corresponding adjacency matrix $A^{\left(v\right)}$ was obtained according to the vth figure (Figure 3c). $A^{\left(v\right)}$ was defined as,

(10) $${\left(A^{\left(v\right)}\right)}_{ij}=\{\begin{array}{l} 1,\;\mathrm{if}\;\mathrm{the}\;\mathrm{drug}\;r_{j}\;\mathrm{is}\;\mathrm{one}\;\mathrm{of}\;\mathrm{the}\;k\;\mathrm{most}\;\mathrm{similar}\;\mathrm{neighbours}\;\\ \;\mathrm{of}\;\mathrm{the}\;\mathrm{drug}\;r_{i}\;\mathrm{based}\;\mathrm{on}\;\mathrm{the}\;\mathrm{vth}\;\mathrm{drug}\;\mathrm{similarity}\;\\ 0,\;\mathrm{otherwise} \end{array}$$

Since drug $r_{i}$ and its neighbour $r_{j}$ are more likely to be associated with a similar group of diseases, a drug-related smoothing term can be created,

(11) $$\begin{array}{l} \frac{1}{2}\overset{4}{\underset{v=1}{\sum }}\overset{N_{r}}{\underset{i,j=1}{\sum }}{\left(A^{\left(v\right)}\right)}_{ij}∥F_{i}-F_{j}{∥}^{2}\\ =\overset{4}{\underset{v=1}{\sum }}\left(Tr\left(F^{T}U^{\left(v\right)}F\right)-Tr\left(F^{T}A^{\left(v\right)}F\right)\right)\;\\ =\overset{4}{\underset{v=1}{\sum }}Tr\left(F^{T}L^{\left(v\right)}F\right)\; \end{array}$$

where $F_{i}$ and $F_{j}$ denote the ith and jth row vectors of F, respectively, and indicate the cases of a potential association of drug $r_{i}$ and $r_{j}$ with all diseases. $U^{\left(v\right)}\in R^{N_{r}\times N_{r}}$ is a diagonal matrix, where ${\left(U^{\left(v\right)}\right)}_{ii}=\sum_{j=1}^{N_{r}}{\left(A^{\left(v\right)}\right)}_{ij}$ and the Laplacian matrix of the vth feature graph is $L^{\left(v\right)}=U^{\left(v\right)}-A^{\left(v\right)}$.

Similarly, the disease $d_{i}$ and its k neighbours are more likely to be associated with similar drugs. Therefore, we established a graph with disease as a node according to disease similarity and obtained the adjacency matrix $A_{d}$ defined as (Figure 3d),

(12) $${\left(A_{d}\right)}_{ij}=\{\begin{array}{l} 1,\;\mathrm{if}\;\mathrm{the}\;\mathrm{disease}\;d_{j}\;\mathrm{is}\;\mathrm{one}\;\mathrm{of}\;\mathrm{the}\;k\;\mathrm{most}\;\\ \;\mathrm{similar}\;\mathrm{neighbours}\;\mathrm{of}\;\mathrm{the}\;\mathrm{disease}\;d_{i}\;\\ 0,\;\mathrm{otherwise} \end{array}$$

Therefore, disease–related regularization items were created as follows,

(13) $$\begin{array}{l} \frac{1}{2}\overset{N_{r}}{\underset{i,j=1}{\sum }}{\left(A_{d}\right)}_{ij}∥{\left(F^{T}\right)}_{i}-{\left(F^{T}\right)}_{j}{∥}^{2}\\ =Tr\left(FU_{d}F^{T}\right)-Tr\left(FA_{d}F^{T}\right)\\ =Tr\left(FL_{d}F^{T}\right)\; \end{array}$$

where ${\left(F^{T}\right)}_{i}$ and ${\left(F^{T}\right)}_{j}\;$were the ith and jth row of $F^{T}$, respectively. They represent the potential association of disease $d_{i}$ and $d_{j}$ with all drugs. $U_{d}\in R^{N_{d}\times N_{d}}$ was a diagonal matrix, ${\left(U_{d}\right)}_{ii}=\sum_{j=1}^{N_{d}}{\left(A_{d}\right)}_{ij}$, and $L^{\left(v\right)}=U^{\left(v\right)}-A^{\left(v\right)}$ was the Laplace matrix of the characteristic graph of the disease. Then, we added a smoothness term to the objective function,

(14) $$\underset{H^{\left(v\right)},W^{\left(v\right)}\geq 0}{\min}∥M⊙{\left(F-Y\right)∥}_{F}^{2}+\alpha_{1}\overset{4}{\underset{v=1}{\sum }}∥X^{\left(v\right)}-W^{\left(v\right)}H^{\left(v\right)}{∥}_{F}^{2}+\alpha_{2}\overset{4}{\underset{w\neq v}{\sum }}DIVE\left(H^{\left(v\right)},H^{\left(w\right)}\right)\;+\alpha_{3}\overset{4}{\underset{v=1}{\sum }}∥F-H^{{\left(v\right)}^{T}}{∥}_{F}^{2}+\alpha_{4}\left(\overset{4}{\underset{v=1}{\sum }}Tr\left(F^{T}L^{\left(v\right)}F\right)+Tr\left(FL_{d}F^{T}\right)\right)$$

where $\alpha_{4}$ adjusts the contribution of the smoothing term.

**Considering the sparsity of drug–disease associations.** The potential associations between drugs and diseases was limited. Thus, drug–disease associations have sparse properties. We used the $l_{1}$-norm to adjust the association matrix for sparse associations. We created the final objective function after adding the sparse item,

(15) $$\underset{H^{\left(v\right)},W^{\left(v\right)}\geq 0}{\min}∥M⊙{\left(F-Y\right)∥}_{F}^{2}+\alpha_{1}\overset{4}{\underset{v=1}{\sum }}∥X^{\left(v\right)}-W^{\left(v\right)}H^{\left(v\right)}{∥}_{F}^{2}+\alpha_{2}\overset{4}{\underset{w\neq v}{\sum }}DIVE\left(H^{\left(v\right)},H^{\left(w\right)}\right)\;+\alpha_{3}\overset{4}{\underset{v=1}{\sum }}∥F-H^{{\left(v\right)}^{T}}{∥}_{F}^{2}+\alpha_{4}\left(Tr\left(\overset{4}{\underset{v=1}{\sum }}F^{T}L^{\left(v\right)}F\right)+Tr\left(FL_{d}F^{T}\right)\right)+\alpha_{5}{∥F∥}_{1}$$

where $\alpha_{5}$ is a regulation parameter.

### 3.4. Optimization

Since the objective Function (15) with the variables F, $H^{\left(v\right)}$ and $W^{\left(v\right)}$ is a non-convex function, it was impractical to derive a global optimal solution. Therefore, we divided the optimization problem into three subproblems and performed iterative optimization, converging each subproblem to a local minimum.

F**-subproblem**. We updated F with fixed $W^{\left(v\right)}$ and $H^{\left(v\right)}$, and the resulting formula contains only the unknown variable F,

(16) $$\begin{array}{ll} minL\left(F\right)=∥M⊙ & {\left(F-Y\right)∥}_{F}^{2}+\alpha_{3}\overset{4}{\underset{v=1}{\sum }}∥F-H^{{\left(v\right)}^{T}}{∥}_{F}^{2}\\ & +\alpha_{4}\left(Tr\left(\overset{4}{\underset{v=1}{\sum }}F^{T}L^{\left(v\right)}F\right)+Tr\left(FL_{d}F^{T}\right)\right)+\alpha_{5}{∥F∥}_{1} \end{array}$$

The item containing the Frobenius norm in Equation (16) was changed to the form of the matrix trace, which can be rewritten as,

(17) $$\begin{array}{ll} L\left(F\right)=Tr(M⊙ & \left(FF^{T}-FY^{T}-YF^{T}+YY^{T}\right))\\ & +\alpha_{3}\overset{4}{\underset{v=1}{\sum }}Tr\left(FF^{T}-FH^{\left(v\right)}-H^{{\left(v\right)}^{T}}F^{T}+H^{{\left(v\right)}^{T}}H^{\left(v\right)}\right)\\ & +\alpha_{4}\left(Tr\left(\overset{4}{\underset{v=1}{\sum }}F^{T}L^{\left(v\right)}F\right)+Tr\left(FL_{d}F^{T}\right)\right)+\alpha_{5}{∥F∥}_{1} \end{array}$$

By setting the derivative of L(F) with respect to *F* to 0, we obtained,

(18) $$2M⊙\left(F-Y\right)+2\alpha_{3}\overset{4}{\underset{v=1}{\sum }}\left(F-H^{{\left(v\right)}^{T}}\right)+2\alpha_{4}\left(\overset{4}{\underset{v=1}{\sum }}\left(U^{\left(v\right)}-A^{\left(v\right)}\right)F+F\left(U_{d}-A_{d}\right)\right)+\alpha_{5}=0$$

where all elements in matrix $B=\left[B_{ij}\right]\in {ℜ}^{N_{r}\times N_{d}}$ are 1. By multiplying both sides of Equation (18) with $F_{ij}$, the following equation was obtained,

(19) $$(2M⊙\left(F-Y\right)+2\alpha_{3}\overset{4}{\underset{v=1}{\sum }}\left(F-H^{{\left(v\right)}^{T}}\right){+2\alpha_{4}\left(\overset{4}{\underset{v=1}{\sum }}\left(U^{\left(v\right)}-A^{\left(v\right)}\right)F+F\left(U_{d}-A_{d}\right)\right)+\alpha_{5}B)}_{ij}F_{ij}=0.$$

We updated *F* according to the coordinate gradient descent Algorithm [38], and derived an updated formula,

(20) $$F_{ij}^{new}\leftarrow F_{ij}\frac{{\left(2M*Y+2\alpha_{3}\sum_{v=1}^{4}H^{{\left(v\right)}^{T}}+2\alpha_{4}\sum_{v=1}^{4}A^{\left(v\right)}F+2a_{4}FA_{d}\right)}_{ij}}{{\left(2M*F+8F+2\alpha_{4}\sum_{v=1}^{4}U^{\left(v\right)}F+2a_{4}FU_{d}+\alpha_{5}B\right)}_{ij}}$$

$H^{\left(v\right)}$**-subproblem**. We updated $H^{\left(v\right)}$ with fixed F and $W^{\left(v\right)}$. The function that only containing the variable $H^{\left(v\right)}$ was as follows,

(21) $$\underset{H^{\left(v\right)}\geq 0}{\min}L\left(H^{\left(v\right)}\right)=\alpha_{1}∥X^{\left(v\right)}-W^{\left(v\right)}H^{\left(v\right)}{∥}_{F}^{2}+\alpha_{2}\overset{4}{\underset{w\neq v}{\sum }}DIVE\left(H^{\left(v\right)},H^{\left(w\right)}\right)+\alpha_{3}\overset{4}{\underset{v=1}{\sum }}∥F-H^{{\left(v\right)}^{T}}{∥}_{F}^{2}.$$

The term of the Frobenius norm in Equation (21) was changed to the form of the matrix trace. Assuming that $\eta_{ij}^{\left(v\right)}$ is the Lagrangian multiplier of constraint $H_{ij}^{\left(v\right)}\geq 0$, and $\eta^{\left(v\right)}=\left[\eta_{ij}{}^{\left(v\right)}\right]$, the resulting Lagrangian function of $H^{\left(v\right)}$ was as follows,

(22) $$\begin{array}{ll} \underset{H^{\left(v\right)}\geq 0}{min}L\left(H^{\left(v\right)}\right)= & \alpha_{1}Tr(X^{\left(v\right)}X^{{\left(v\right)}^{T}}-X^{\left(v\right)}H^{{\left(v\right)}^{T}}W^{{\left(v\right)}^{T}}\\ & -W^{\left(v\right)}H^{\left(v\right)}X^{{\left(v\right)}^{T}}+W^{\left(v\right)}H^{\left(v\right)}H^{{\left(v\right)}^{T}}W^{{\left(v\right)}^{T}})+\alpha_{2}\overset{4}{\underset{w\neq v}{\sum }}Tr\left(H^{\left(v\right)}H^{{\left(w\right)}^{T}}\right)\\ & +\alpha_{3}Tr\left(FF^{T}-FH^{\left(v\right)}-H^{{\left(v\right)}^{T}}F^{T}+H^{{\left(v\right)}^{T}}H^{\left(v\right)}\right)+Tr\left(\eta^{\left(v\right)}H^{\left(v\right)}\right). \end{array}$$

By setting the derivative of $L\left(H^{\left(v\right)}\right)$ with respect to $H^{\left(v\right)}$ to 0, we obtained,

(23) $$\alpha_{1}\left(2W^{{\left(v\right)}^{T}}W^{\left(v\right)}H^{\left(v\right)}-2W^{{\left(v\right)}^{T}}X^{\left(v\right)}\right)+\alpha_{2}\overset{4}{\underset{w\neq v}{\sum }}H^{\left(w\right)}+\alpha_{3}\left(2H^{\left(v\right)}-2F^{T}\right)+\eta^{\left(v\right)}=0$$

According to the KTT condition $\eta_{ij}^{\left(v\right)}H_{ij}^{\left(v\right)}=0$, we derived the following formula,

(24) $${\left(2\alpha_{1}W^{{\left(v\right)}^{T}}W^{\left(v\right)}H^{\left(v\right)}-2\alpha_{1}W^{{\left(v\right)}^{T}}X^{\left(v\right)}+\alpha_{2}\overset{4}{\underset{w\neq v}{\sum }}H^{\left(w\right)}+2\alpha_{3}H^{\left(v\right)}-2\alpha_{3}F^{T}\right)}_{ij}H_{ij}^{\left(v\right)}=0$$

Then we obtained the updated formula for $H^{\left(v\right)}$,

(25) $${\left(H_{ij}^{\left(v\right)}\right)}^{new}\leftarrow H_{ij}^{\left(v\right)}\frac{{\left(2\alpha_{1}W^{{\left(v\right)}^{T}}X^{\left(v\right)}+2\alpha_{3}F^{T}\right)}_{ij}}{{\left(2\alpha_{1}W^{{\left(v\right)}^{T}}W^{\left(v\right)}H^{\left(v\right)}+\alpha_{2}\sum_{w\neq v}^{4}H^{\left(w\right)}+2\alpha_{3}H^{\left(v\right)}\right)}_{ij}}\;$$

$W^{\left(v\right)}$**-subproblem**. By using fixed F and $H^{\left(v\right)}$, we could update $W^{\left(v\right)}$. The subproblem with $W^{\left(v\right)}$ as the only variable was as follows,

(26) $$\underset{W^{\left(v\right)}\geq 0}{\min}L\left(W^{\left(v\right)}\right)=\alpha_{1}∥X^{\left(v\right)}-W^{\left(v\right)}H^{\left(v\right)}{∥}_{F}^{2}\;$$

Then, we changed the term containing the Frobenius norm in Equation (26) to the form of the matrix trace, and let $\beta^{\left(v\right)}=\left[\beta_{ij}{}^{\left(v\right)}\right]$ be the Lagrangian multiplier with the constraint $W^{\left(v\right)}\geq 0$. The resulting Lagrangian function for $W^{\left(v\right)}$ was as follows,

(27) $$\begin{array}{ll} \underset{W^{\left(v\right)}\geq 0}{min}L\left(W^{\left(v\right)}\right)= & \alpha_{1}(X^{\left(v\right)}X^{{\left(v\right)}^{T}}-2X^{\left(v\right)}H^{{\left(v\right)}^{T}}W^{{\left(v\right)}^{T}}-W^{\left(v\right)}H^{\left(v\right)}X^{{\left(v\right)}^{T}}+W^{\left(v\right)}H^{\left(v\right)}H^{{\left(v\right)}^{T}}W^{\left(v\right)})\\ & +Tr\left(\beta^{\left(v\right)}W^{\left(v\right)}\right) \end{array}$$

By setting the derivative of $L\left(W^{\left(v\right)}\right)$ to $W^{\left(v\right)}$ to 0, we created the following formula,

(28) $$2\alpha_{1}W^{\left(v\right)}H^{\left(v\right)}H^{{\left(v\right)}^{T}}-2\alpha_{1}X^{\left(v\right)}H^{{\left(v\right)}^{T}}+\beta^{\left(v\right)}=0$$

Similarly, according to the KTT condition $\beta_{ij}^{\left(v\right)}W_{ij}^{\left(v\right)}=0$, we derived,

(29) $${\left(2\alpha_{1}W^{\left(v\right)}H^{\left(v\right)}H^{{\left(v\right)}^{T}}-2\alpha_{1}X^{\left(v\right)}H^{{\left(v\right)}^{T}}\right)}_{ij}W_{ij}^{\left(v\right)}=0$$

Therefore, the updated formula for $W^{\left(v\right)}$ was as follows,

(30) $${\left(W_{ij}^{\left(v\right)}\right)}^{new}\leftarrow W_{ij}^{\left(v\right)}\frac{{\left(X^{\left(v\right)}H^{{\left(v\right)}^{T}}\right)}_{ij}}{{\left(W^{\left(v\right)}H^{\left(v\right)}H^{{\left(v\right)}^{T}}\right)}_{ij}}\;$$

We solve $F,\;H^{\left(v\right)}$, and $W^{\left(v\right)}$ iteratively by using the above updating rules. Finally, $F_{ij}\;$is regarded as the estimated association score between drug $r_{i}\;$and disease$\;d_{j}$ (Algorithm 1).

**Algorithm 1** DivePred algorithm for predicting the potential drug-disease associations.**Input:** A drug-disease association matrix $Y\in {ℜ}^{N_{r}\times N_{d}}$ and the drugs character matrix$\;X_{1}\in {ℜ}^{881\times N_{r}},\;X_{2}\in {ℜ}^{1426\times N_{r}},\;X_{3}\in {ℜ}^{4447\times N_{r}},\;X_{4}\in {ℜ}^{N_{d}\times N_{r}}$.**Output:** Drug-disease association score matrix F, where $F_{ij}$ is the association score for drug $r_{i}\;$and disease $d_{j}$.
Randomly initialize the elements in $F,H^{\left(v\right)},W^{\left(v\right)}$ (1≤v≤4) with the values between 0 and 1.While $L\left(F^{\left(v\right)},H^{\left(v\right)},W^{\left(v\right)}\right)$ not converged do Fix $W^{\left(v\right)}$ and $H^{\left(v\right)}$, along with an update for F, using the rule: $$F_{ij}^{new}\leftarrow F_{ij}\cdot \frac{{\left(2M*Y+2\alpha_{3}\sum_{v=1}^{4}H^{{\left(v\right)}^{T}}+2\alpha_{4}\sum_{v=1}^{4}A^{\left(v\right)}F+2a_{4}FA_{d}\right)}_{ij}}{{\left(2M*F+8F+2\alpha_{4}\sum_{v=1}^{4}U^{\left(v\right)}F+2a_{4}FU_{d}+\alpha_{5}B\right)}_{ij}}$$ For v=1 to 4  Fix F and $W^{\left(v\right)}$, along with an update for $H^{\left(v\right)}$, using the rule: $${\left(H_{ij}^{\left(v\right)}\right)}^{new}\leftarrow H_{ij}^{\left(v\right)}\frac{{\left(2\alpha_{1}W^{{\left(v\right)}^{T}}X^{\left(v\right)}+2\alpha_{3}F^{T}\right)}_{ij}}{{\left(2\alpha_{1}W^{{\left(v\right)}^{T}}W^{\left(v\right)}H^{\left(v\right)}+\alpha_{2}\sum_{w\neq v}^{4}H^{\left(w\right)}+2\alpha_{3}H^{\left(v\right)}\right)}_{ij}}$$ End for For v=1 to 4  Fix F and $H^{\left(v\right)}$, along with an update for $W^{\left(v\right)}$, using the rule: $${\left(W_{ij}^{\left(v\right)}\right)}^{new}\leftarrow W_{ij}^{\left(v\right)}\frac{{\left(X^{\left(v\right)}H^{{\left(v\right)}^{T}}\right)}_{ij}}{{\left(W^{\left(v\right)}H^{\left(v\right)}H^{{\left(v\right)}^{T}}\right)}_{ij}}\;$$ End for End While


## 4. Conclusions

A method based on non-negative matrix factorization, DivePred, was developed to infer the potential associations between drugs and diseases. DivePred captures a variety of information on each drug, including four kinds of drug features and specific features associated with different aspects of the drugs. Meanwhile, it also captures disease–disease similarities and drug–disease associations. The projection of multiple kinds of drug features, along with the drugs and diseases neighbour information, was completely integrated to enhance the inference of drug–disease associations. An iterative algorithm was developed to estimate drug–disease association scores that can be used to prioritize disease candidates for each drug. DivePred outperforms other methods in AUCs and AUPRs. For biologists, DivePred is very useful because more real drug–disease associations were included in DivePred’s top-ranking candidate list. Case studies on five drugs demonstrated that DivePred could detect potentially new indications for drugs. DivePred can serve as a prioritization tool to screen the potential candidates for subsequent discovery of real drug–disease associations through biological validation.

## Figures and Tables

**Table 1:** Area under ROC curve (AUC) values of 15 drugs using DivePred and other methods.

Drug Name	AUC DivePred	TL_HGBI	MBiRW	LRSSL	SCMFDD
ampicillin	**0.944**	0.751	0.932	0.962	0.895
cefepime	**0.976**	0.910	0.970	0.971	0.914
cefotaxime	**0.992**	0.917	0.929	0.950	0.953
cefotetan	**0.996**	0.808	0.918	0.948	0.848
cefoxitin	**0.979**	0.890	0.912	0.979	0.894
ceftazidime	**0.985**	0.845	0.931	0.936	0.922
ceftizoxime	0.797	0.960	0.961	0.923	**0.962**
ceftriaxone	0.907	0.945	0.898	**0.955**	0.811
ciprofloxacin	**0.957**	0.811	0.813	0.928	0.820
doxorubicin	**0.949**	0.487	0.921	0.727	0.460
erythromycin	**0.962**	0.827	0.887	0.918	0.764
itraconazole	**0.952**	0.445	0.877	0.845	0.730
levofloxacin	**0.975**	0.943	0.975	0.964	0.872
moxifloxacin	0.794	0.812	0.948	0.957	0.932
ofloxacin	**0.958**	0.902	0.943	0.904	0.774
Average AUC	**0.926**	0.683	0.837	0.838	0.726

The bold values indicate the higher AUCs.

**Table 2:** Area under precision–recall curve (AUPR) values of 15 drugs using DivePred and other methods.

Drug Name	AUPR DivePred	TL_HGBI	MBIRW	LRSSL	SCMFDD
ampicillin	**0.189**	0.032	0.023	0.285	0.068
cefepime	**0.744**	0.163	0.315	0.625	0.054
cefotaxime	**0.770**	0.071	0.292	0.283	0.105
cefotetan	**0.486**	0.054	0.197	0.512	0.059
cefoxitin	**0.580**	0.151	0.394	0.286	0.065
ceftazidime	0.675	0.032	0.201	0.488	**0.694**
ceftizoxime	**0.647**	0.212	0.244	0.455	0.096
ceftriaxone	0.409	0.056	0.223	**0.673**	0.077
ciprofloxacin	**0.425**	0.082	0.118	0.280	0.064
doxorubicin	0.164	0.005	0.051	**0.180**	0.004
erythromycin	**0.425**	0.023	0.038	0.144	0.022
itraconazole	0.188	0.006	**0.253**	0.042	0.008
levofloxacin	0.504	0.136	0.071	**0.539**	0.098
moxifloxacin	**0.565**	0.049	0.065	0.384	0.088
ofloxacin	**0.378**	0.091	0.130	0.201	0.078
Average AUC	**0.200**	0.013	0.043	0.117	0.014

The bold values indicate the higher AUPRs.

**Table 3:** Results of Wilcoxon test on DivePred and four other contrast methods for 763 drugs.

*p*-Value Between DivePred and Another Method	TL_HGBI	MBiRW	LRSSL	SCMFDD
*p*-value of ROC curve	5.631 × 10^−42^	7.181 × 10^−156^	3.735 × 10^−78^	6.596 × 10^−73^
*p*-value of PR curve	1.332 × 10^−21^	2.635 × 10^−32^	1.562 × 10^−16^	8.452 × 10^−29^

**Table 4:** The top 15 related candidate diseases for acetaminophen, ciprofloxacin, doxorubicin, hydrocortisone, and ampicillin.

Drug Name	Rank	Disease Name	Description	Rank	Disease Name	Description
*Acetaminophen*	1	Osteoarthritis	CTD	9	Arthritis	DrugBank
	2	Arthritis, Rheumatoid	CTD	10	Pain, Postoperative	CTD
	3	Inflammation	CTD	11	Rheumatic Fever	PubChem
	4	Dysmenorrhea	inferred candidate by 1 literature	12	Arthritis, Gouty	CTD
	5	Arthritis, Juvenile Rheumatoid	DrugBank	13	Premenstrual Syndrome	DrugBank
	6	Gout	DrugBank	14	Menorrhagia	unconfirmed
	7	Spondylitis, Ankylosing	Clinicaltrials	15	Rheumatic Diseases	Clinicaltrials
	8	Bursitis	literature [30]			
*Ciprofloxacin*	1	Salmonella Infections	CTD	9	Pyelonephritis	CTD
	2	Streptococcal Infections	DrugBank	10	Bacterial Infections	CTD
	3	Bronchitis	CTD	11	Serratia Infections	DrugBank
	4	Pneumonia, Bacterial	CTD	12	Tuberculosis, Pulmonary	CTD
	5	Chlamydia Infections	CTD	13	Plague	CTD
	6	Gram-Negative Bacterial Infections	CTD	14	Brucellosis	PubChem
	7	Enterobacteriaceae Infections	CTD	15	Chlamydiaceae Infections	PubChem
	8	Soft Tissue Infections	CTD			
*Doxorubicin*	1	Leukemia, Myeloid, Acute	CTD	9	Rhabdomyosarcoma	CTD
	2	Precursor Cell Lymphoblastic Leukemia-Lymphoma	CTD	10	Histiocytosis	Clinicaltrials
	3	Carcinoma, Non-Small-Cell Lung	PubChem	11	Trophoblastic Neoplasms	DrugBank
	4	Mycosis Fungoides	PubChem	12	Stomach Neoplasms	CTD
	5	Leukemia, Lymphocytic, Chronic, B-Cell	inferred candidate by 14 literatures	13	Hodgkin Disease	CTD
	6	Head and Neck Neoplasms	CTD	14	Melanoma	CTD
	7	Sarcoma, Kaposi	CTD	15	Leukemia, Myelogenous, Chronic, BCR-ABL Positive	DrugBank
	8	Leukemia, Lymphoid	CTD			
*Hydrocortisone*	1	Asthma	CTD	9	Shock, Septic	CTD
	2	Rhinitis, Allergic, Perennial	DrugBank	10	Acne Vulgaris	unconfirmed
	3	Dermatitis	PubChem	11	Rosacea	CTD
	4	Skin Diseases	CTD	12	Addison Disease	CTD
	5	Pruritus	PubChem	13	Hyperhidrosis	literature [31]
	6	Keratosis	inferred candidate by 1 literature	14	Hematologic Diseases	inferred candidate by 1 literature
	7	Hypersensitivity	inferred candidate by 7 literatures	15	Pityriasis Rosea	unconfirmed
	8	Psoriasis	PubChem			
*Ampicillin*	1	Proteus Infections	CTD	9	Osteomyelitis	Clinicaltrials
	2	Streptococcal Infections	CTD	10	Impetigo	unconfirmed
	3	Septicemia	DrugBank	11	Serratia Infections	CTD
	4	Pneumonia, Bacterial	CTD	12	Peritonitis	CTD
	5	Bone Diseases, Infectious	PubChem	13	Bacterial Infections	CTD
	6	Staphylococcal Skin Infections	DrugBank	14	Enterobacteriaceae Infections	DrugBank
	7	Wound Infection	CTD	15	Cellulitis	CTD
	8	Pseudomonas Infections	PubChem			

## Supplementary Materials

Supplementary materials can be found at https://www.mdpi.com/1422-0067/20/17/4102/s1.
